# Using Google Trends and Wikipedia to Investigate the Global Public’s Interest in the Pancreatic Cancer Diagnosis of a Celebrity

**DOI:** 10.3390/ijerph20032106

**Published:** 2023-01-24

**Authors:** Vincenza Gianfredi, Daniele Nucci, Mariateresa Nardi, Omar Enzo Santangelo, Sandro Provenzano

**Affiliations:** 1 Department of Biomedical Sciences for Health, University of Milan, Via Pascal, 36, 20133 Milan, Italy; 2 Nutritional Support Unit, Veneto Institute of Oncology IOV-IRCCS, Via Gattamelata, 64, 35128 Padua, Italy; 3 Regional Health Care and Social Agency of Lodi, Azienda Socio Sanitaria Territoriale di Lodi (ASST Lodi), Piazza Ospitale 10, 26900 Lodi, Italy; 4 Local Health Unit of Trapani, ASP Trapani, 91100 Trapani, Italy

**Keywords:** pancreatic cancer, Google Trends, medical informatics computing, Wikipedia, Italy

## Abstract

A cross-sectional study was designed to assess the impact of a celebrity’s announcement of having been diagnosed with pancreatic cancer on the volume of cancer-related research on the Internet. Global searches were carried out on Google Trends (GT) for the period from 1 January 2004 to 20 November 2022 (since data prior to 2004 were not available) using the search words Tumore del Pancreas (pancreatic cancer), Tumore neuroendocrino (neuroendocrine tumor), and Fedez (the name of a popular Italian rapper). The frequency of specific page views for Fedez, Tumore del pancreas, and Tumore neuroendocrino was collected via Wikipedia Trends data. Statistical analyses were carried out using the Pearson correlation coefficient (r). The GT data revealed a strong correlation (r = 0.83) while the Wikipedia Trends data indicated a moderate correlation (r = 0.37) for Tumore neuroendocrino and Tumore del pancreas. The search peaks for the GT and Wikipedia pages occur during the same time period. An association was found between the celebrity’s announcement of his pancreatic cancer diagnosis and the volume of pancreatic-cancer-related online searches. Our findings demonstrate that media events and media coverage of health-related news can raise people’s curiosity and desire for health information.

## 1. Introduction

Pancreatic cancer has one of the worst prognoses of all cancers, with a five-year survival rate of just 11% [1]. One reason for this dismal prognosis is that 90% of pancreatic cancers are frequently diagnosed at an advanced stage of the disease, when the tumor is no longer operable, as a result of late clinical expression with systemic metastases in > 50% of patients [2,3]. Despite the low overall incidence of pancreatic cancer (5.7 per 100,000 person-years), the most recent Global Burden of Diseases, Injuries, and Risk Factors Study found that the number of deaths, incident cases, and disability-adjusted life years attributable to the disease has more than doubled worldwide from 1990 to 2017 [4]. It is anticipated that pancreatic cancer will be the second leading cause of cancer-related mortality in the United States by 2030 [5], with the highest incidence and mortality rates seen in high-income countries [4]. In recent years, traditional chemotherapy and radiotherapy for pancreatic cancer have been improved, first-line and second-line palliative treatments have been developed, and adjuvant treatments have also been used in clinical trials to improve the efficacy of alleviating symptoms and disease outcome. However, due to the low five-year survival rate, new treatment methods, such as targeted therapy and immunotherapy, need to be investigated [6]. Despite the considerable variability in cost estimates, which are driven by the type of tumor and cancer stage, the direct costs of pancreatic cancer are in the upper band relative to that of other cancer types, while the indirect costs are also substantial and mainly attributed to high mortality. The high costs impose a heavy economic burden on society and reduce patients’ health-related quality of life (HRQoL) [7]. Given the impact of cancer on global health, the goal of public health policies is to improve global survival through three pillars: health promotion, timely diagnoses, and comprehensive treatment and supportive care. Therefore, an evaluation of the global public’s interest in this disease is crucial. Patients with access to cancer information are more likely to take an active role in disease management, as it prepares them for treatment and helps them cope with associated adverse effects, reduces anxiety and depression, increases treatment satisfaction, improves communication with family, and improves quality of life [8].

In recent decades, technological advances, such as mobile applications for patient-centered care coordination, have aided in the empowerment of cancer illness awareness in the population. Patients can turn to the Internet for information on disorders, medications, and treatments; therefore, this type of search traffic data could be a very important source for analyzing the trends of various health-related topics and could be a method for gauging public interest in them. Although this field, known as “infodemiology”, is still in its infancy, it can be envisioned that its implications for big data analysis will be positive, as has been demonstrated for infectious diseases [9,10,11,12], Internet-based surveillance systems [13], and revelations regarding the effects of the recent COVID-19 pandemic on mental health [14]. There is a paucity of data in the scientific literature about pancreatic cancer-related digital searching behavior, despite the use of Google Trends (GT) and Wikipedia searches, which may be superior to other data systems in terms of their low cost, transparency, simplicity, and reproducibility across a variety of domains. In March 2022, Fedez, a very popular Italian rapper, disclosed on his Instagram profile that he had undergone surgery for pancreatic cancer. Using GT and Wikipedia searches and analyzing them, the purpose of this study was to investigate how Internet public searches can be leveraged to quantify the impact of a disease on public opinion in the case of a famous individual.

## 2. Materials and Methods

The research employed a cross-sectional study design. Data on Internet searches were obtained from GT, which is based on Google Search, the most widely used Internet search engine that analyzes the popularity of search topics in Google using graphs to compare the search volumes of different queries over time and across different geographical locations [15]. GT and Wikipedia data were extracted on 20 November 2022. Searches on Google Trends were carried out between 1 January 2004 and 20 November 2022 (from inception; data before 1 January 2004 are not available) using the search words Tumore del Pancreas (pancreatic cancer), Tumore neuroendocrino (neuroendocrine tumor), and Fedez (the name of a popular Italian rapper). Three partly overlapping time frames were extracted. The first was from 1 January 2004 to 20 November 2022, with data aggregated monthly; the second was from 5 December 2021 to 20 November 2022 (date of data extraction), with data aggregated on a weekly basis; and the third was from 3 October 2021 to 30 October 2022, with data aggregated weekly for comparison with Wikipedia page search data over the same period. The relative search volume (RSV) changes according to the selected period, since it is a relative index. The file was downloaded in “.CSV” format. GT produces a relative search volume (RSV) scaled to the highest search proportion week or month, which is computed as the percentage of queries concerning a particular term for a specific location and time period, in which 100 is the maximum value and 0 is the minimum value. Thus, RSV allows for direct comparisons of search volumes across search terms. From Wikipedia [16] it is possible to determine the number of times a specific page is viewed by users; data were extracted as daily data and aggregated on a weekly or monthly basis to enable comparison and to view the data together with those of GT or between them. The following data were extracted: the number of page views from July 2015 (from inception; data prior to July 2015 were not available) to October 2022 (date of extraction was on 20 November; Wikipedia data were available up to October 2022) for Fedez, Tumore del pancreas (pancreatic cancer), and Tumore neuroendocrino (neuroendocrine tumor). The words “Tumore pancreatico” and “Tumore neuroendocrine” were reported as specific words in the “Disease category”, which identify the specific topic in GT. “Fedez” is a specific word from the “Rapper” section, which specifies a specific person in GT. Similarly, the three above-mentioned words identify specific pages in Wikipedia database. The same approach was used in previous studies in which Internet search peaks were assessed in relation to celebrities’ cancer diagnoses [17,18].

Statistical analyses were performed using the Pearson correlation coefficient (r). By rule of thumb, a correlation is high if r > 0.7, moderate if the value of r is between 0.3 and 0.7, and weak if r < 0.3 [19]. Google search terms were correlated with each other and then with Wikipedia, first considering the source of data availability, 2004 for Google Trends and 2015 for Wikipedia, then extracting and considering data from the previous year. A similar correlational analysis was performed in previous studies in which a spatial-temporal assessment was conducted [20,21]. The significance level for statistical analyses was set at 0.05. Data were analyzed using the STATA statistical software, version 14 [22].

## 3. Results

The results revealed a temporal correlation between the Google Trends searches and Wikipedia pages viewed. Table 1 displays the correlation between the search terms Fedez, Tumore del pancreas, and Tumore neuroendocrino in Google Trends. The correlation was high for Tumore neuroendocrino and Tumore del pancreas (r = 0.83), while the correlation was moderate for Fedez and Tumore del pancreas (r = 0.57) and for Fedez and Tumore neuroendocrino (r = 0.46). 

Table 2 illustrates the correlation between the Wikipedia pages viewed for Fedez, Tumore del pancreas, and Tumore neuroendocrino. The correlation was moderate for Tumore neuroendocrino and Tumore del pancreas (r = 0.37), while the correlation was weak for Fedez and Tumore del pancreas (r = 0.19) and Fedez and Tumore neuroendocrino (r = 0.06, not statistically significant).

Table 3 depicts the correlation between the search terms Fedez, Tumore del pancreas, and Tumore neuroendocrino in Google trends. The correlation was high for Tumore neuroendocrino and Tumore del pancreas (r = 0.99), while the correlation was moderate for Fedez and Tumore del pancreas (r = 0.61) and Fedez and Tumore neuroendocrino (r = 0.62). 

Table 4 presents the correlation between the Wikipedia pages viewed for Fedez, Tumore del pancreas, and Tumore neuroendocrino. The correlation was moderate for Tumore neuroendocrino and Tumore del pancreas (r = 0.65), while the correlation was weak for Fedez and Tumore del pancreas (r = 0.19) and for Fedez and Tumore neuroendocrino (r = 0.12). In general, the data from the last year have stronger correlations (see Table 2 and Table 4) than the correlations from the beginning of the data extraction possibility (see Table 1 and Table 3). 

**Figure 1:**
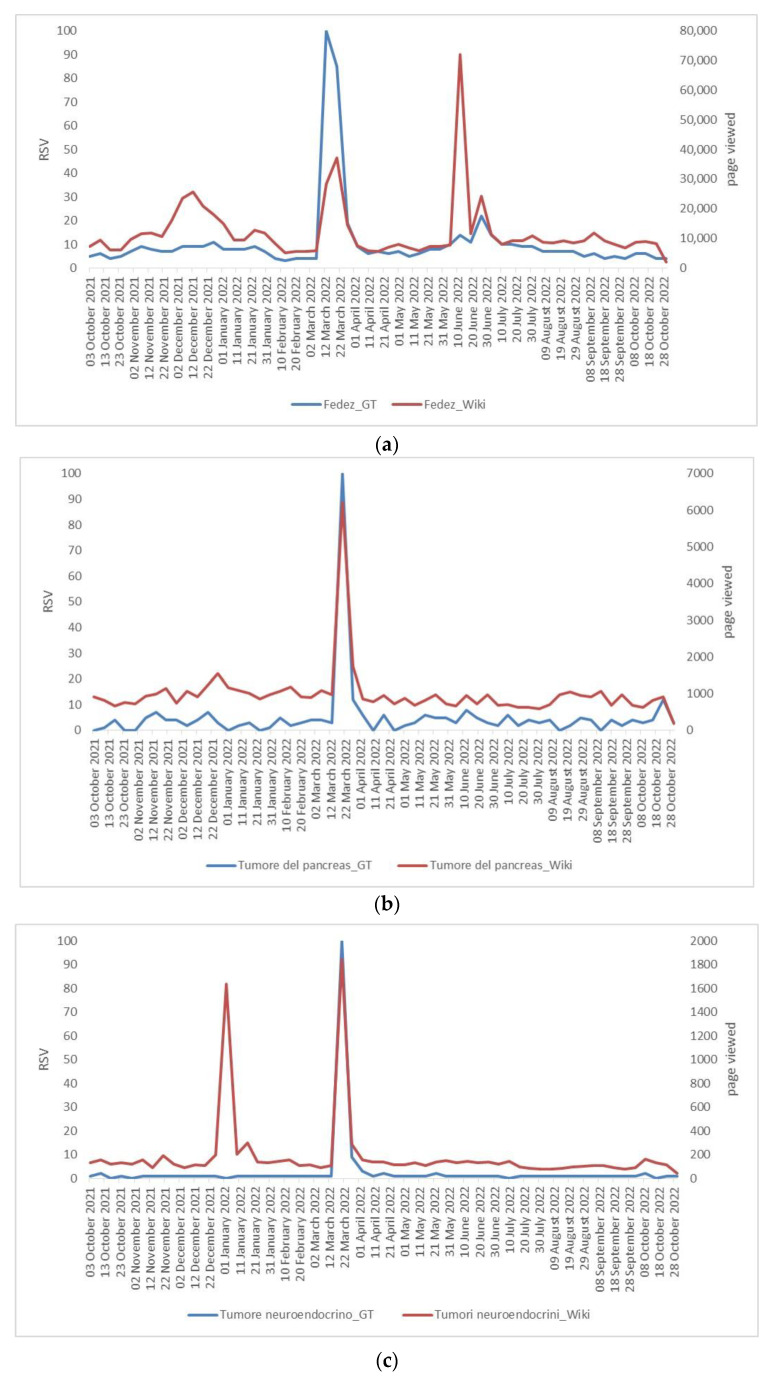
Comparison of Google Trends and Wikipedia search term curves: (**a**) comparing the word Fedez; (**b**) pancreatic cancer—Tumore del pancreas in Italian; (**c**) neuroendocrine cancer—Tumore neuroendocrino in Italian. Period analyzed from 3 October 2021 to 30 October 2022; data were aggregated weekly.

**Figure 2:**
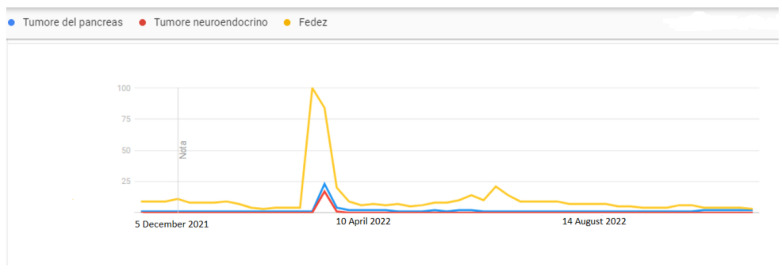
Temporal correlation between Google Trends-based query volumes for “Tumore del Pancreas” (pancreatic cancer), “Tumore neuroendocrino” (neuroendocrine cancer), and “Fedez” in Italy from 5 December 2021 to 20 November 2022, using the option to extract last year’s data on Google Trends.

**Figure 3:**
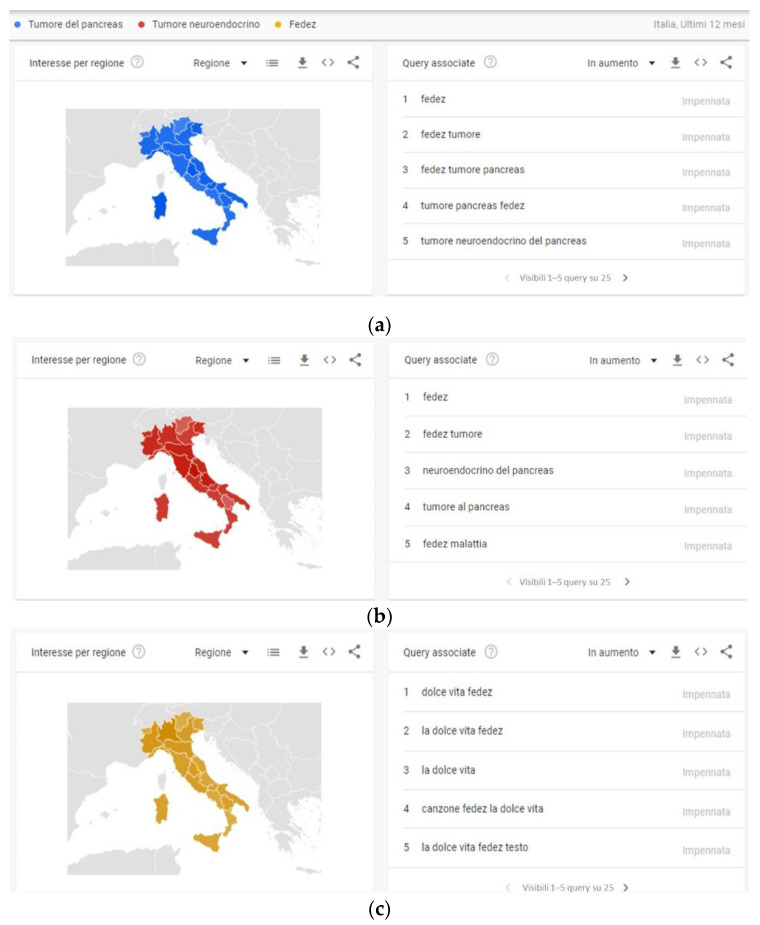
The top five queries on Google Trends connected with the “Fedez” (**a**), “Tumore del pancreas”; (**b**) pancreatic cancer in English, and “Tumore neuroendocrino”; (**c**) neuroendocrine cancer in English search terms.

As shown in Figure 1 and Figure 2, the peak search periods for Google Trends and Wikipedia pages occur during the same time period, because Internet users searched for the topics more frequently in the same period. As shown in Figure 3, “Fedez” is the search word that is most associated with pancreatic cancer and neuroendocrine cancer in Google Trends.

## 4. Discussion

This manuscript assesses online information for pancreatic cancer trends. More specifically, we tested the hypothesis that a celebrity’s announcement of having been diagnosed with pancreatic cancer may influence the behavior of Internet users, leading to an increase in searches for cancer-related information. Our results confirmed this hypothesis, verifying that news in the media associated with the announcement of a celebrity’s illness, such as pancreatic cancer, arouses people’s interest. This heightened interest is reflected in a rise in the volume of Internet searches for disease-related information. In particular, our study revealed an association between the disease’s announcement and an increase in search volumes, as measured by both Google Trends and Wikipedia. We investigated the two because Google is a search engine commonly used to find general news and information, while Wikipedia is a free online encyclopedia frequently used to research obscure or unfamiliar topics. In this perspective, it is plausible that after public personalities announce their illness, people first conduct a Google search to confirm the news and possibly the type of disease, and then consult Wikipedia to expand their knowledge on the subject. 

Our findings are consistent with the only previous study conducted on the topic. This study conducted by Noar et al. in 2013 examined the impact of celebrities’ cancer announcements on Google search volumes in the United States [23]. In this study, the authors found an association between the announcement of a celebrity’s pancreatic cancer diagnosis or death and an increase in search query results for some, but not all, of the personalities investigated. In this case, search volumes may be affected by the level of media coverage and the public figure’s notoriety, as well as the time period during which the announcements were made. The above-mentioned study was published in 2013, and some of the announcements considered were even older, potentially indicating a lower level of Internet availability among the general population and, consequently, a lower use of the Internet to seek information, particularly health-related information. However, the study conducted by Foroughi et al. revealed a rise in people’s interest in searching for information about cancer [24]. When cancer types were assessed, breast cancer maintained a steadily high ranking, although pancreatic cancer garnered increasing attention throughout the study period (2004–2015).

Novel data streams, and in particular infodemiology, are a relatively new area of research that is progressively being used in numerous and different fields of application. Several studies have been conducted on the potential role of this data in infectious disease surveillance, namely influenza [9], COVID-19 [11], arbovirus [10], tropical and subtropical infectious diseases [25], and pertussis [26], but also chronic diseases, such as mental health [14] and rheumatoid arthritis [27], as well as lifestyle factors, such as diet [28]. Previous research on infectious diseases has revealed an association between the number of cases recorded in surveillance systems and the number of Internet searches on symptomatology [9,10,11,25,27]. In addition, the authors found that the Internet was typically accessed one week before the first case was flagged in the surveillance systems, suggesting that there is a time lag between the onset of symptoms, Internet searches, and notifications on traditional surveillance systems [9].

### 4.1. Implications for Public Health Policies and Practice

These data are relevant because they provide an overview of the general public’s behavior with regard to searching for online health information [29]. In particular, they can provide information on the time of year when people are particularly interested in certain health topics, are more receptive to receiving information, and, hopefully, in adopting a new lifestyle, or simply becoming more aware of preventive strategies, the importance of early diagnosis, and the need for prompt treatment. Additionally, these data provide information regarding which topics are considered relevant by the general population [30]. From a public health perspective, this may represent an important opportunity to disseminate up-to-date and accurate information on the prevention, diagnosis, and treatment of specific diseases, if the target population deems it relevant. This information could boost the effectiveness of public health communication campaigns by facilitating the timely delivery of engaging content. In a previous study, for instance, Nucci et al., found a seasonality trend in searching for information on diets in Italy [28]. A similar seasonality pattern in search volumes was also observed in previous studies assessing Internet information-seeking trends on exposure to risk factors (tobacco use [31] or sun exposure [32]) and associated cancer (lung cancer or skin cancer, respectively), or between disease(s) awareness day(s) and specific disease(s) [33].

Based on the evidence accumulated to date, the dynamic nature of our society, and the Internet’s ease of access, infodemiological assessments are a novel approach that may provide virtually real-time data useful for informing public health initiatives [34]. In this regard, and based on our findings, institutional communication should be implemented utilizing all available tools, including institutional websites, institutional social networks, portals, educational tutorials/videos, forums, and smartphone applications [35]. In fact, the vast reach of the Internet enables the rapid dissemination of (mis)information to a wide audience. Therefore, institutions that fail or neglect to provide scientifically valid information presented in layman’s terms contribute to the dissemination of incomplete or deliberately misleading information [35]. The public health workforce needs to be proficient in e-communication in in layman’s terms in order to tackle and meet the public health challenges of the new millennium [36]. Our results also highlighted the need to capitalize on celebrity notoriety and strengthen alliances targeted at disseminating valid, straightforward, and easily understandable health-related information.

### 4.2. Strengths and Limitations

Before generalizing our results, certain limitations must be considered. Firstly, this study should be viewed as a pilot study on the impact of a celebrity’s announcement regarding his pancreatic cancer diagnosis and the ensuing volume of cancer-related searches on the Internet. To the best of our knowledge, this is the first study undertaken in Italy to examine the pattern of pancreatic cancer search volumes. We used data from both GT and Wikipedia; however, these do not represent all of the options available to Internet users seeking online information. Other search engines, such as Yahoo! or Bing, were not considered, nor were data from social networks assessed in this study. Nevertheless, previous research indicates that Google is used by more than 80% of Internet users worldwide [37]. Although GT does not provide information about user characteristics, which makes it impossible to characterize people who are searching for specific topics, these novel data can still provide scientists and policymakers with a wide range of opportunities, given that the analysis of novel data streams has opened a new research strand that could be applied to a variety of subject areas, including acute diseases; emerging, re-emerging, or “old” infectious diseases; and chronic diseases, such as cancer. Infodemiological assessments may also assist policymakers by providing data on the attitudes and behaviors of the general population when searching for health-related information. However, this type of analysis does not evaluate the content of the sought information, and consequently their validity and accuracy. This is an important element that might limit the application of these results. In order to contextualize and interpret results in light of the above-mentioned limitation, this new approach should be supported and supplemented by conventional analytical methods, such as questionnaires or medical data assessments.

## 5. Conclusions

To conclude, we identified a correlation between a celebrity’s announcement of a pancreatic cancer diagnosis and the volume of pancreatic cancer-related Internet searches generated. Our findings demonstrate that media events and media coverage of health-related news can arouse people’s curiosity about and desire for health information. Therefore, authoritative institutional websites and web pages containing up-to-date and accurate information are fundamental for counteracting fake news and accurately informing the public, particularly regarding sought-after topics. Lastly, collaborating with celebrities could increase public awareness of relevant public health issues and preventive measures.

## Figures and Tables

**Table 1:** Pearson’s correlation coefficient (r) for Google Trends search terms in Italy from January 2004 to November 2022.

		Fedez_GT	Tumore del Pancreas_GT (Pancreatic Cancer in GT)	Tumore Neuroendocrino_GT (Neuroendocrine Tumor in GT)
Fedez_GT	r	1		
	*p*-value	-		
Tumore del pancreas_GT (pancreatic cancer in GT)	r	0.57	1	
	*p*-value	<0.001	-	
Tumore neuroendocrino_GT (neuroendocrine tumor in GT)	r	0.46	0.83	1
	*p*-value	<0.001	<0.001	-

GT: Google Trends.

**Table 2:** Pearson’s correlation coefficient (r) for Wikipedia searches in Italy from July 2015 to October 2022.

		Fedez_Wiki	Tumore del Pancreas_Wiki (Pancreatic Cancer in Wiki)	Tumore Neuroendocrino_Wiki (Neuroendocrine Tumor in Wiki)
Fedez_Wiki	r	1		
		-		
Tumore del pancreas_Wiki (pancreatic cancer in Wiki)	r	0.19	1	
	*p*-value	0.07	-	
Tumore euroendocrine_Wiki (neuroendocrine tumor in Wiki)	r	0.06	0.37	1
	*p*-value	0.60	<0.001	-

Wiki: Wikipedia.

**Table 3:** Pearson’s correlation coefficient (r) for Google Trends search terms in Italy from 5 December 2021 to 20 November 2022.

		Fedez_GT	Tumore del Pancreas_GT (Pancreatic Cancer in GT)	Tumore Neuroendocrino_GT (Neuroendocrine Tumor in GT)
Fedez_GT	r	1		
	*p*-value	-		
Tumore del pancreas_GT (pancreatic cancer in GT)	r	0.61	1	
	*p*-value	<0.001	-	
Tumore neuroendocrino_GT (neuroendocrine tumor in GT)	r	0.62	0.99	1
	*p*-value	<0.001	<0.001	-

GT: Google Trends.

**Table 4:** Pearson’s correlation coefficient (r) for Wikipedia searches in Italy from 1 October 2021 to 31 October 2022.

		Fedez_Wiki	Tumore del Pancreas_Wiki (Pancreatic Cancer in Wiki)	Tumore Neuroendocrino_Wiki (Neuroendocrine Tumor in Wiki)
Fedez_Wiki	r	1		
	*p*-value	-		
Tumore del pancreas_Wiki (pancreatic cancer in Wiki)	r	0.193	1	
	*p*-value	<0.001	-	
Tumore neuroendocrino_Wiki (neuroendocrine tumor in Wiki)	r	0.12	0.65	1
	*p*-value	0.019	<0.001	-

Wiki: Wikipedia.

## Data Availability

Not applicable.

## References

[1] American Cancer Society (2022). Cancer Facts & Figures 2022. American Cancer Society, Atlanta. https://www.cancer.org/content/dam/cancer-org/research/cancer-facts-and-statistics/annual-cancer-facts-and-figures/2022/2022-cancer-facts-and-figures.pdf.

[2] Kommalapati A., Tella S.H., Goyal G., Ma W.W., Mahipal A. (2018). Contemporary Management of Localized Resectable Pancreatic Cancer. Cancers.

[3] Siegel R.L., Miller K.D., Fuchs H.E., Jemal A. (2021). Cancer Statistics, 2021. CA Cancer J. Clin..

[4] GBD2017 Pancreatic Cancer Collaborators (2019). The global, regional, and national burden of pancreatic cancer and its attributable risk factors in 195 countries and territories, 1990–2017: A systematic analysis for the Global Burden of Disease Study 2017. Lancet Gastroenterol. Hepatol..

[5] Rahib L., Smith B.D., Aizenberg R., Rosenzweig A.B., Fleshman J.M., Matrisian L.M. (2014). Projecting cancer incidence and deaths to 2030: The unexpected burden of thyroid, liver, and pancreas cancers in the United States. Cancer Res..

[6] Cao D., Song Q., Li J., Jiang Y., Wang Z., Lu S. (2021). Opportunities and challenges in targeted therapy and immunotherapy for pancreatic cancer. Expert Rev. Mol. Med..

[7] Hernandez D., Wagner F., Hernandez-Villafuerte K., Schlander M. (2022). Economic Burden of Pancreatic Cancer in Europe: A Literature Review. J. Gastrointest. Cancer.

[8] Chua G.P., Tan H.K., Gandhi M. (2018). What information do cancer patients want and how well are their needs being met?. Ecancermedicalscience.

[9] Gianfredi V., Santangelo O.E., Provenzano S. (2021). Correlation between flu and Wikipedia’s pages visualization. Acta Biomed.

[10] Provenzano S., Gianfredi V., Santangelo O.E. (2021). Insight the data: Wikipedia’s researches and real cases of arboviruses in Italy. Public Health.

[11] Santangelo O.E., Provenzano S., Gianfredi V. (2021). Infodemiology of flu: Google trends-based analysis of Italians’ digital behavior and a focus on SARS-CoV-2, Italy. J. Prev. Med. Hyg..

[12] Santangelo O.E., Gianfredi V., Provenzano S. (2022). Wikipedia searches and the epidemiology of infectious diseases: A systematic review. Data Knowl. Eng..

[13] Santangelo O.E., Grigis D., Giordano D., Armetta F., Firenze A. (2020). Can Google Trends and Wikipedia help traditional surveillance? A pilot study on measles. Acta Biomed.

[14] Gianfredi V., Provenzano S., Santangelo O.E. (2021). What can internet users’ behaviours reveal about the mental health impacts of the COVID-19 pandemic? A systematic review. Public Health.

[15] (2022). Google Trends. Explore What the World is Searching. Google, United States. https://trends.google.it/trends/?geo=IT.

[16] (2022). Wikipedia. Analysis of Page Views. Wikipedia, United States. https://tools.wmflabs.org/pageviews.

[17] Kaleem T., Malouff T.D., Stross W.C., Waddle M.R., Miller D.H., Seymour A.L., Zaorsky N.G., Miller R.C., Trifiletti D.M., Vallow L. (2019). Google Search Trends in Oncology and the Impact of Celebrity Cancer Awareness. Cureus.

[18] Ayers J.W., Althouse B.M., Noar S.M., Cohen J.E. (2014). Do celebrity cancer diagnoses promote primary cancer prevention?. Prev. Med..

[19] Mukaka M.M. (2012). Statistics corner: A guide to appropriate use of correlation coefficient in medical research. Malawi Med. J..

[20] Metcalfe D., Price C., Powell J. (2010). Media coverage and public reaction to a celebrity cancer diagnosis. J. Public Health.

[21] Naik H., Johnson M.D.D., Johnson M.R. (2021). Internet Interest in Colon Cancer Following the Death of Chadwick Boseman: Infoveillance Study. J. Med. Internet. Res..

[22] StataCorp (2015). Stata Statistical Software.

[23] Noar S.M., Ribisl K., Althouse B.M., Willoughby J., Ayers J.W. (2013). Using Digital Surveillance to Examine the Impact of Public Figure Pancreatic Cancer Announcements on Media and Search Query Outcomes. JNCI Monogr..

[24] Foroughi F., Lam A.K.-Y., Lim M.S., Saremi N., Ahmadvand A., Zeng D., Neto O.L., Mackey T., Ginossar T., Dreyer N. (2016). “Googling” for Cancer: An Infodemiological Assessment of Online Search Interests in Australia, Canada, New Zealand, the United Kingdom, and the United States. JMIR Cancer.

[25] Gianfredi V., Bragazzi N.L., Nucci D., Martini M., Rosselli R., Minelli L., Moretti M. (2018). Harnessing Big Data for Communicable Tropical and Sub-Tropical Disorders: Implications from a Systematic Review of the Literature. Front. Public Health.

[26] Gianfredi V., Bragazzi N., Mahamid M., Bisharat B., Mahroum N., Amital H., Adawi M. (2018). Monitoring public interest toward pertussis outbreaks: An extensive Google Trends–based analysis. Public Health.

[27] Mahroum N., Bragazzi N.L., Sharif K., Gianfredi V., Nucci D., Rosselli R., Brigo F., Adawi M., Amital H., Watad A. (2018). Leveraging Google Trends, Twitter, and Wikipedia to Investigate the Impact of a Celebrityʼs Death from Rheumatoid Arthritis. Am. J. Clin. Oncol..

[28] Nucci D., Santangelo O.E., Nardi M., Provenzano S., Gianfredi V. (2021). Wikipedia, Google Trends and Diet: Assessment of Temporal Trends in the Internet Users’ Searches in Italy before and during COVID-19 Pandemic. Nutrients.

[29] Bragazzi N.L., Barberis I., Rosselli R., Gianfredi V., Nucci D., Moretti M., Salvatori T., Martucci G., Martini M. (2016). How often people google for vaccination: Qualitative and quantitative insights from a systematic search of the web-based activities using Google Trends. Hum. Vaccines Immunother..

[30] Bragazzi N.L., Gianfredi V., Villarini M., Rosselli R., Nasr A., Hussein A., Martini M., Behzadifar M. (2018). Vaccines Meet Big Data: State-of-the-Art and Future Prospects. From the Classical 3Is (“Isolate–Inactivate–Inject”) Vaccinology 1.0 to Vaccinology 3.0, Vaccinomics, and Beyond: A Historical Overview. Front. Public Health.

[31] Zhang Z., Zheng X., Zeng D.D., Leischow S.J. (2015). Information Seeking Regarding Tobacco and Lung Cancer: Effects of Seasonality. PLoS ONE.

[32] Bloom R., Amber K.T., Hu S., Kirsner R. (2015). Google Search Trends and Skin Cancer. JAMA Dermatol..

[33] Murray G., O’Rourke C., Hogan J., Fenton J. (2016). Detecting internet search activity for mouth cancer in Ireland. Br. J. Oral Maxillofac. Surg..

[34] Gianfredi V., Grisci C., Nucci D., Parisi V., Moretti M. (2018). Communication in health. Recenti. Prog. Med..

[35] Gianfredi V., Odone A., Fiacchini D., Rosselli R., Battista T., Signorelli C. (2019). Trust and reputation management, branding, social media management nelle organizzazioni sanitarie: Sfide e opportunità per la comunità igienistica italiana. J. Prev. Med. Hyg..

[36] Gianfredi V., Balzarini F., Gola M., Mangano S., Carpagnano L.F., Colucci M.E., Gentile L., Piscitelli A., Quattrone F., Scuri S. (2019). Leadership in Public Health: Opportunities for Young Generations Within Scientific Associations and the Experience of the “Academy of Young Leaders”. Front. Public Health.

[37] Malik S. (2014). A Comparative Study of two major Search Engines: Google and Yahoo. Orient. J. Comput. Sci. Technol..

